# Achromobacter xylosoxidans Bacteremia in a Liver Transplant Patient: A Case Report and Literature Review

**DOI:** 10.7759/cureus.26048

**Published:** 2022-06-17

**Authors:** Munsef Barakat, Jamal Sajid

**Affiliations:** 1 Internal Medicine Residency Program, Medical Education, Hamad General Hospital, Doha, QAT; 2 Medicine, Hamad General Hospital, Doha, QAT

**Keywords:** achromobacter xylosoxidans, immunocompromised, septic shock, liver transplantation, gram negative bacteremia

## Abstract

Since the first isolation of Achromobacter xylosoxidans, it has been increasingly recognized as an opportunistic pathogen. It is an aerobic Gram-negative bacillus mainly found in aquatic environments. It has been reported to cause nosocomial infections, especially in immunocompromised patients. This organism has a unique susceptibility to antimicrobials, being resistant to most commonly used cephalosporins and aminoglycosides, with susceptibility to piperacillin/tazobactam and most carbapenems. In this case, we report a case of a 60-year-old female with a history of liver transplantation, who developed nosocomial Achromobacter xylosoxidans bacteremia complicated by septic shock, multi-organ failure, and death.

## Introduction

Achromobacter xylosoxidans is a motile, aerobic Gram-negative bacillus that has been linked to severe infections in humans, mainly in immunocompromised individuals. Achromobacter xylosoxidans was first isolated and described by Yabuuchi and Ohyama in 1971 [1]. Although the first isolation by Yabuuchi and Ohyama was from ear discharges of patients with otitis media, it is widely believed that the organism is mainly found in aquatic environments and causes infections in immunocompromised individuals. Over the years, Achromobacter species have been linked to multiple outbreaks of nosocomial infections, especially in ICU settings. An example of such an outbreak is the cluster of cases of nosocomial Achromobacter infection in Kerala, India, which was found to be caused by contaminated furosemide ampoules [2]. Infections caused by the organism can vary, from pneumonia, bacteremia, meningitis, and device-related infections. Bacteremia is considered to be the most common. In one retrospective study, all Achromobacter species were susceptible to meropenem and piperacillin-tazobactam with resistance to aminoglycosides, aztreonam, and most of the cephalosporins [3].

## Case presentation

A 60-year-old female presented with a history of type 2 diabetes mellitus and chronic kidney disease secondary to diabetes. She had a history of liver transplantation at the age of 45 due to liver cirrhosis secondary to chronic Hepatitis C virus infection, treated successfully with a sustained virologic response.

The patient presented with vomiting and diarrhea for five days. The diarrhea was watery and occurred around 5-6 times per day, with no hematemesis or bleeding per rectum. The patient did not report any abdominal pain or fever. On the day of admission, the patient felt generalized weakness and dizziness followed by a fall but no loss of consciousness or abnormal movement. At home, she was taking Tacrolimus 0.5 mg daily. She was followed up regularly by the hepatology outpatient clinic and was taking her medications regularly.

On examination, the patient was clinically stable, fully conscious and oriented, afebrile, and had dry oral mucosa. Abdomen, respiratory, and cardiovascular examinations were unremarkable. Neurological examination was normal, and no focal findings were found.

On admission, vital signs were as follows: 120/64 mmHg, heart rate (HR) 107 bpm, temperature(T) 36.7 ℃, respiratory rate (RR) 17 breaths per minute, and oxygen saturation was 98% on ambient air.

Blood tests on admission showed evidence of acute kidney injury (worsening from her baseline) and high anion gap metabolic acidosis, with liver enzymes within normal limits (Table 1).

**Table 1:** Laboratory findings on the day of admission and on the day of deterioration. ALT: Alanine aminotransferase; AST: Aspartate transaminase.

Laboratory	Day 1	After deterioration
Hemoglobin	9.5 g/dl	8.6 g/dl
WBC	2.2 x10^3/uL	2.7 x10^3/uL
Creatinine	155 umol/L	268 umol/L
Urea	8 mmol/L	11.9 mmol/L
Bicarbonate	13 mmol/L	9 mmol/L
PH	7.36	7.2
Lactate	3.4 mmol/L	13 mmol/L
C-reactive protein	6.5 mg/L	99.5 mg/L
Procalcitonin	-	2.44 ng/mL
ALT	20 U/L	143 U/L
AST	49 U/L	1100 U/L
Tacrolimus Level (Trough)	5.0 ng/ml	-

She was admitted to the medial floor as a case of possible gastroenteritis and dehydration. Blood cultures, blood for cytomegalovirus (CMV) polymerase chain reaction (PCR), and stool for Clostridium difficile toxins along with stool culture were sent. She was started on IV ceftriaxone and metronidazole along with IV fluid resuscitation. Ceftriaxone and metronidazole were discontinued within 48 hours of admission as there was no evidence of infection.

Her stool workup revealed a positive Clostridium difficile positive PCR but was negative for Clostridium toxins; however, because the patient continued to have diarrhea, oral vancomycin for Clostridium difficile treatment was initiated.

Over the next few days, the patient's renal function continued to deteriorate, although she clinically remained stable and her diarrhea improved.

On the seventh day of the hospital admission, the patient became hypotensive and drowsy. New sets of blood cultures were sent and started empirically on piperacillin/tazobactam with the impression of the development of sepsis. As blood pressure failed to respond to IV fluid boluses and the patient showed evidence of hypoperfusion evident by a change in sensorium along with elevated serum lactate, she was admitted to the medical ICU and started on norepinephrine and vasopressin infusion. Blood culture revealed Achromobacter xylosoxidans, which was sensitive to piperacillin/tazobactam and meropenem (Table 2).

**Table 2:** Blood culture result and sensitivity. MIC: Minimal inhibitory concentration.

	Achromobacter xylosoxidans
Drug	MIC Interpretation
Amikacin	Resistant
Cefepime	Resistant
Ceftazidime	Intermediate
Ciprofloxacin	Resistant
Gentamicin	Resistant
Levofloxacin	Resistant
Meropenem	Sensitive
Piperacillin/Tazobactam	Sensitive
Trimethoprim/Sulfamethoxazole	Sensitive

As the patient's condition did not improve, antibiotics escalated to meropenem. Her condition deteriorated, and she developed multi-organ failure. She required intubation and mechanical ventilation. Unfortunately, she died 48 hours after ICU admission.

## Discussion

Achromobacter xylosoxidans is a recognized pathogen in immunocompromised patients. Achromobacter xylosoxidans has low virulence but has been known to cause nosocomial infection, particularly in high-risk individuals [3,4]. In addition, many risk factors have been identified to increase the risk of infection by Achromobacter xylosoxidans, including immunosuppression, prolonged hospitalization, malignancy, neutropenia, and the presence of a central venous catheter [5]. Many healthcare-associated infections and outbreaks of Achromobacter xylosoxidans have been reported previously due to contaminated medical equipment and medications [2,6], and surgical site infections causing delayed surgical wounds have been described [7].

In the reviewed articles, the most common feature among the patients is an underlying malignancy. Infective endocarditis typically occurs in patients with valve replacement, although one case of infective endocarditis was reported in a healthy immunocompetent patient with a native valve [8]. One case report described a patient with pulmonary tuberculosis and Achromobacter xylosoxidans bacteremia, which was complicated by septic shock and death [9]. Table 3 highlights previous case reports of Achromobacter xylosoxidans.

**Table 3:** Previous publications describing patients with Achromobacter xylosoxidans bacteremia. M: Male; F: female; IC; Immunocompromised; CVD: Cardiovascular disease; IMD; Invasive medical device; BAV: Bioprosthetic aortic valve; PM: Pacemaker; CABG: Coronary artery bypass graft; AF: Atrial fibrillation; CKD: Chronic kidney disease; PVD: Peripheral vascular disease; ESRD; End-stage renal disease; CHF: Chronic heart failure; COPD: Chronic obstructive pulmonary disease; TIA: Transient ischemic attack; HTN: Hypertension; CVC: Central venous catheter; CAD: Coronary artery disease; ALL: Acute lymphocytic leukemia; HM: Hematological malignancy; SM: Solid malignancy; UTI: Urinary tract infection; AVF: Arterio-venous fistula. * One patient developed more than one positive clinical isolate and may develop more than one hospital-acquired infection; ** Correctional cardiac surgery: interatrial and interventricular communication corrected at 11 months of age and aortic coarctation correction at age 10; *** The author concluded that the infected pleural effusion likely represents hematogenous dissemination; **** An environmental investigation concluded that a contaminated atomizer was the source of the outbreak.

Publication	Patients No.	Age (mean/range)	Gender (no.)	Underlying disease	Focus (no.)	Bacteremia
Yilmaz G et al. [10]	1	70 years	Male	Pancreatic cancer /Post ERCP	Biliary tract	Yes
Marion-Sanchez K et al. [11]	66 (69 cases of hospital-acquired infection) *	Mean age 61.5 years	M (42), F (24)	IC 29, CVD 16, respiratory disease 7, HM 7, SM 15, IMD 55	Pneumonia (26), bacteremia (23), intra-abdominal infection (7), UTI (7), mediastinitis (3), wound infection (1), pharyngitis (2)	23 patients (33.3%)
Sawant AC et al. [12]	1	62 years	Female	BAV, PM, AF, CKD, CHF, COPD,PVD	Infective endocarditis	Yes
de Castro RL et al. [8]	1	19 years	Male	Heart surgery**	Infective endocarditis	Yes
Storey A et al. [13]	1	79 years	Female	TIA, HTN, AF	Infective endocarditis	Yes
Manfredi R et al. [14]	7	Range 24-38 years	F (4), M (3)	HIV (7), CVC (2), Ex-IV drug users (6)	Respiratory tract (4)	Yes (all patients)
Dupon M [15]	1	35 years	Female	Liver transplant recipient	Biliary tract	Yes
Mandell WF et al. [9]	1	58 years	Male	Pulmonary tuberculosis	Lungs	Yes
Legrand C and Anaissie E [4]	10	15-70 years	M (5), F (5)	All patient had either: HM/SM, neutropenia (4)	CVC (4) GI tract (3), cholecystitis (1) pneumonia(2) , CVC-related infection (4)	Yes ( all patients)
Tokuyasu H et al. [16]	1	86 years	Female	Prosthetic aortic valve replacement	Infective endocarditis	Yes
Shimamura T et al. [17]	1	78 years	Male	ESRD, CAD, CABG, HTN	Bacteremia, infected pleural effusion***	Yes
Lee W-S et al. [18]	1	72 years	Female	ESRD, Hepatocellular carcinoma	Infected AVF	Yes
Dai J et al. [19]	1	20 years	Male	ALL, neutropenia	Cellulitis	Yes
Tena D et al. [5]	4	Range 63-80 years	F (3) , M (1)	ESRD on long term intravascular catheter	Catheter-related infection****	Yes (all patients)
Turgutalp K et al. [20]	1	67 years	Male	DM, CHF, ESRD, CVC	CVC-related infection	Yes

Antibiotic sensitivity was consistent in the literature, with most strains resistant to cephalosporins, aztreonam, and aminoglycosides and being susceptible to piperacillin/tazobactam, meropenem, imipenem, and sulfamethoxazole-trimethoprim, with variable resistance to fluoroquinolones [3,10].

Our patient had multiple risk factors for developing severe infection given her age, comorbidities such as diabetes mellitus, chronic kidney disease, history of liver transplantation, and use of immunosuppressants along with hospitalization. It is worth mentioning that the initial blood cultures on admission were negative, thus concluding that the bacteremia was acquired during her hospital stay. Although the patient was started on the appropriate antibiotics even before the culture result, unfortunately, she developed multi-organ failure. She died within a few days of admission to the medical ICU.

## Conclusions

In reviewing the available literature, it was concluded that Achromobacter xylosoxidans is an opportunistic pathogen that can cause serious nosocomial infection, especially in immunocompromised patients, which can be fatal. In our case report, the patient developed severe septic shock and did not respond to appropriate antimicrobial therapy, given her significant medical history. Therefore, awareness of this organism needs to be raised, especially given its antibiotic resistance pattern, which must be kept in mind when starting empirical therapy.
